# Improvement in the prediction of the translation initiation site through balancing methods, inclusion of acquired knowledge and addition of features to sequences of mRNA

**DOI:** 10.1186/1471-2164-12-S4-S9

**Published:** 2011-12-22

**Authors:** Lívia Márcia Silva, Felipe Carvalho de Souza Teixeira, José Miguel Ortega, Luis Enrique Zárate, Cristiane Neri Nobre

**Affiliations:** 1 Departamento de Ciência da Computação - Pontifícia Universidade Católica de Minas Gerais, Brazil; 2 Departamento de Ciência da Computação - Universidade Federal de São João del-Rei, Brazil; 3 Departamento of Bioquímica and Imunologia - Universidade Federal de Minas Gerais, Brazil

## Abstract

**Background:**

The accurate prediction of the initiation of translation in sequences of mRNA is an important activity for genome annotation. However, obtaining an accurate prediction is not always a simple task and can be modeled as a problem of classification between positive sequences (protein codifiers) and negative sequences (non-codifiers). The problem is highly imbalanced because each molecule of mRNA has a unique translation initiation site and various others that are not initiators. Therefore, this study focuses on the problem from the perspective of balancing classes and we present an undersampling balancing method, M-clus, which is based on clustering. The method also adds features to sequences and improves the performance of the classifier through the inclusion of knowledge obtained by the model, called InAKnow.

**Results:**

Through this methodology, the measures of performance used (accuracy, sensitivity, specificity and adjusted accuracy) are greater than 93% for the *Mus musculus* and *Rattus norvegicus* organisms, and varied between 72.97% and 97.43% for the other organisms evaluated: *Arabidopsis thaliana*, *Caenorhabditis elegans*, *Drosophila melanogaster*, *Homo sapiens*, *Nasonia vitripennis*. The precision increases significantly by 39% and 22.9% for *Mus musculus* and *Rattus norvegicus*, respectively, when the knowledge obtained by the model is included. For the other organisms, the precision increases by between 37.10% and 59.49%. The inclusion of certain features during training, for example, the presence of ATG in the upstream region of the Translation Initiation Site, improves the rate of sensitivity by approximately 7%. Using the M-Clus balancing method generates a significant increase in the rate of sensitivity from 51.39% to 91.55% (*Mus musculus*) and from 47.45% to 88.09% (*Rattus norvegicus*).

**Conclusions:**

In order to solve the problem of TIS prediction, the results indicate that the methodology proposed in this work is adequate, particularly when using the concept of acquired knowledge which increased the accuracy in all databases evaluated.

## Background

Transcription and translation are the means by which the cells interpret and express their genetic information [1]. Only part of the transcribed sequences carries information to codify proteins (CDS -CoDing Sequence). In other words, even though mRNA can be translated in its entirety, only a section of this mRNA is translated into amino acid [2]. Therefore, given a molecule of mRNA, a central problem of molecular biology is to determine whether it contains CDS and thereafter to discover which protein will be codified. The region of the mRNA sequence where the initiation of the protein synthesis process occurs is called the Translation Initiation Site (TIS).

Control of the initiation of translation is one of the most important processes in the regulation of genetic expression [3]. Thus, determining the TIS is not a trivial task; it is of great relevance for genetic inference. A high level of accuracy of prediction could be useful for a better understanding of the protein obtained from the sequences of nucleotides [4].

Normally, translation begins in the first ATG of the mRNA molecule that has an appropriate context [5], but can begin in a different codon [6]. Depending on the position of the synthesis initiation in the mRNA strand, the triplet of nucleotides selected for the synthesis can vary, also altering the amino acids generated. The lack of knowledge about the preservative features in the process of identifying the initiation of translation makes the prediction of the TIS a complex task. For this reason, computational methods which identify patterns can be used with the aim of extracting the implicit knowledge involved in this process [2]. Since 1982, the prediction of the TIS has been studied extensively using biological methods, statistics and computational techniques [1]. Initially, statistical methods were exploited with the aim of discovering patterns in positive sequences. The pioneering work of Kozak [5], a statistical analysis of the sequences of 211 mRNAs of eukaryotic cells, revealed that some positions in the sequences of mRNAs, relative to the TIS, are very stable, determining the Kozak consensus [5], gcc[a/g]ccatg[g], where there is a predominance of these nucleotides in positions -3 and +4.

Another statistical analysis was conducted by Cavener *et. al *[7] on the Start codon (the codon which initiates translation) and the Stop codons (codons which finalize translation), and an algorithm was developed to analyze the frequency of the nucleotides and the multiple positions of the nucleotides. In the work developed by Kozak [5], a proportion of 79% of Adenine (A) in position -3 was identified (and 18% of G) while Cavener *et. al *[7], using 2,595 vertebrate sequences, obtained a 58% probability of A being in the aforementioned position.

Nakagawa *et. al *[3] conducted comparative analyses between 47 species, including animals, fungi, plants and protists, revealing the existence of consensus for different species. Based on this analysis, the following regions of consensus were identified: the presence of a purine (A or G) in position -3, the presence of A or C in position -2, the presence of C in position +5. The position -3 had already been discovered by Kozak [5] and was confirmed by this study.

Different computational methods have been applied to the prediction of the TIS including Artificial Neural Networks (ANN) [8,9], Support Vector Machines (SVM) [2,10,11] and the Gaussian Model [12]. Utilizing Artificial Neural Networks, Stormo *et. al *[8] classified the sequences of Escherichia coli using codification of 4 bits (A=1000, C=0100, G=0010, T=0001) and windows of 51, 71 and 101 nucleotides centered on ATG. Pedersen and Nielsen [9], however, trained Artificial Neural Networks using a database of vertebrates which was processed to obtain the correspondent sequences of mRNA. Of these sequences, only those with the TIS annotated and with at least 10 nucleotides in the upstream region and at least 150 in the downstream region were selected. The resultant base had 13,502 ATGs, 3,312 (24.5%) being TIS and the other 10,190 (75.4%) being non-TIS. In this study, windows of 13, 33, 53, 73, 93, 113, 133, 153, 173 and 203 nucleotides centered on ATG were used. The codification used was the same as that of Stormo *et. al *[8] - binary of 4 bits. Pedersen and Nielsen [9] obtained sensitivity, specificity and accuracy of 78%, 87% and 85%, respectively. The authors also conducted an analysis of the sequences to reveal that features are important for distinguishing TIS from non-TIS. It was discovered that position -3 is crucial in the identification of the TIS and this corroborates with the other studies cited.

Hatzigeorgiou [6] also used ANN to classify sequences of human cDNA, achieving accuracy of 94%. The author utilized two modules: *consensus-ANN* (analyses the immediate neighborhood of the TIS candidate) and *coding-ANN* (evaluates the upstream and downstream regions of the candidate). The *consensus-ANN* module evaluates the TIS candidate and its most immediate neighborhood through a window of 12 nucleotides. The sequences were extracted from positions -7 to +5 and the binary codification of 4 bits was used. The *coding-ANN* module evaluates the upstream and downstream regions of the TIS candidate and operates with windows of 54 nucleotides. The final method is the integration of the modules where scores are calculated for each ATG of the molecule and the first ATG which offers a score above 0.2 is considered the TIS of the molecule.

Using SVM, Zien *et. al *[10] achieved accuracy of 88.1% for the same database as Pedersen and Nielsen [9]. The authors also used the same size of window (203 nucleotides) and the same codification. They showed how to obtain improvements using a new kernel function called *locality-improved* kernel with a small window in each position. The *locality-improved* kernel emphasizes correlations between the positions in the sequence that are close to each other and a size of 3 nucleotides upstream and downstream is empirically determined as optimum. In other words, the modification was to favor local correlations between nucleotides while dependencies between nucleotides in distant positions were considered of little importance or nonexistent. With this kernel function, the authors obtained sensitivity, specificity and accuracy of 69.9%, 94.1% and 88.1%, respectively.

At a later date, Zien *et. al *[10] improved these results through a more sophisticated kernel function known as the *Salzberg* kernel. The *Salzberg* kernel is essentially a conditional probabilistic model of the positions of dinucleotides. Using this kernel, the authors obtained an accuracy of 88.6% for the same database. Li *et. al *[11] utilized two new proposals for the identification of the TIS. Firstly, they introduced a class of new kernels based on string edit distance, called *edit kernels*, to be used with SVM. According to the authors, the edit kernels are simple and have significant and probabilistic biological interpretations. Next, they converted the downstream region of an ATG into a sequence of amino acids before applying SVM. They demonstrated that the approach they adopted is significantly better (sensitivity = 99.92%, specificity = 99.82% and accuracy = 99.9% for the database used by Pedersen and Nielsen [9]).

Nobre, Ortega and Braga [2] conducted experiments to discover the TIS using 12 nucleotides in the upstream and downstream regions, in addition to SVM with simple kernel functions. Inspired by a study conducted on the frequency of triplets of positive and negative sequences, they presented a new codification methodology. Instead of individually codifying each nucleotide, the codification was done per triplet, with a sliding window of size 3. The authors obtained a 50% reduction in the number of entries. In order to balance the data, they used the Smote algorithm [13] to replicate minority class samples. The authors worked with bases of five organisms extracted from the RefSeq database [14]: *Danio rerio*, *Drosophila melanogaster*, *Homo sapiens*, *Mus musculus* and *Rattus norvegicus*, under six levels of inspection. Tzanis *et. al *[15] developed a methodology for the prediction of the TIS, called MANTIS, with three main components: *Consensus*, *Coding Region classification*, and *ATG Location*. The *Coding Region Classification* component involves training a model to classify whether or not the ATG of a sequence is the TIS. They utilized features selected from previous studies [1,4] and PGA (Principal Component Analysis) to obtain the lowest number of non-correlated features, since many are correlated to each other. The *Consensus* component uses Markov rules which capture not only the probability of occurrence of a nucleotide in a determined position, but also how the occurrence of a base interferes with the occurrence of another in the region close to the ATG (between positions -7 and +5). The ATG *location* component is considered a new model, being based on the location of the ATG in the sequence in accordance with the *Ribosome Scanning Model* (RSM) described by Kozak [5,16]. The final stage of MANTIS is the fusion of the decision of the components, the output being the estimated probability of an ATG being a TIS instead of a simple true/false decision. For the prediction, four classification algorithms were used: Naive Bayes, C4.5, K-nearest neighbor and SVM, obtaining an average accuracy and adjusted accuracy of 98.03% and 94.28%, respectively.

Tikole and Sankararamakrishnan [17] used ANN with two hidden layers to predict the TIS in sequences of human mRNA in which there is a week Kozak context. The authors stated that the translation initiation site has a weak Kozak context if purine and guanine are absent in positions -3 and +4, respectively. They obtained sensitivity of 83% and specificity of 73%.

In contrast to other authors, Zeng *et. al *[18] created an algorithm with the aim of constructing representative, dependable and readily available databases free from redundancy in order to facilitate the evaluation of the efficiency of the algorithms used for predicting the TIS. To prepare these databases, they considered three different features: the molecular weight (MW), the isoelectric point (IP) and the hydrophobicity index (HI) profile.

Saeys, Abeel, Degroeve and Peer [19] evaluated the performance of several TIS recognition methods at the genomic level, and compared them to state-of-the-art models for TIS prediction in transcript data. The authors concluded that the simple methods largely outperform the complex ones at the genomic scale, and proposed a new model for TIS recognition at the genome level that combines the strengths of these simple models.

Sparks and Brendel [20] demonstrated that improvements in statistically-based models for TIS prediction can be achieved by taking the class of each potential start-methionine into account, pending certain testing conditions. They developed the MetWAMer package for TIS prediction and demonstrated that the proposed model based on perceptron is suitable for the TIS identification task.

Having identified that the problem of predicting the TIS is highly imbalanced and that the oversampling methods, which have already been used in the present context, significantly increase computational complexity, this study proposes an undersampling class balancing method, M-Clus. This is particularly important for large databases where oversampling techniques are not viable as they significantly increase the size of the databases involved.

In addition to the balancing method, this study also investigates the integration of features into positive and negative sequences, attempting to increase the measures of performance.

Finally, a methodology for the inclusion of acquired knowledge (InAKnow) by the classifier is proposed, where, from the model obtained by training using upstream region sequences and the TIS, the sequences of the downstream region are first classified and later included in new training. This methodology increases the rate of precision of all the evaluated databases.

This paper is organized as follows: Firstly, the “Methods” Section shows all the steps used in this study for the prediction of TIS. To test the proposed methodology the organisms *Mus musculus* and *Rattus norvegicus* have been used as a reference. The “Results and Discussion” Section presents the results obtained by the proposed methodology for these two organisms. Once defined, the best configuration was tested with larger databases such as *Arabidopsis thaliana*, *Caenorhabditis elegans*, *Drosophila melanogaster* and *Homo sapiens*. This is detailed in the “Validation of the methodology with other databases” Section. The “Comparison with other TIS prediction tools” Section provides a comparison between some existing tools for predicting TIS and the methodology proposed in this study. Finally, the “Conclusions” Section presents the final considerations.

## Methods

This section describes the methodology used to develop the proposed TIS prediction model, namely: description of the database used, the form of extraction of the positive and negative sequences from the mRNA, balancing methods, the classifier used, the inclusion of features, incorporation of the knowledge acquired by the classifier, the measures of performance and the validation process used.

### Database

Since the proposed method requires a large amount of testing (multiple window sizes, features, etc.), it was initially tested with the smaller databases, *Mus musculus* and *Rattus Norvegicus*, and then expanded to organisms which have a larger amount of mRNA: *Arabidopsis thaliana*, *Caenorhabditis elegans*, *Drosophila melanogaster* and *Homo sapiens*. All databases were extracted from the public database RefSeq [14] and relate to the organisms under the *reviewed* inspection level already evaluated by Nobre, Ortega and Braga [2].

### Extraction of the positive and negative sequences

In order to use the SVM classifier, positive (TIS) and negative (non-TIS) sequences were extracted through an implemented tool, PredictTIS [21], with variations of windows of the following sizes: -8+8 (8 nucleotides in the upstream and downstream regions, respectively), -12+12, -20+20, -30+30, -40+40, -50+50, -60+60, -10+50, -50+10, -10+30, -10+20, -8+30, -12+20 e -12+30. Initially, experimental tests were conducted with windows of symmetrical size, for example -12+12. However, tests with asymmetrical windows proved to be more efficient. The executable file PredictTIS is available for download from [21].

**Figure 1:**
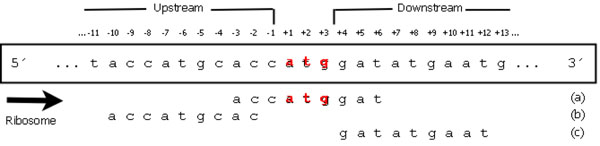
**Extraction of sequences and ribosome scanning model (RSM).** The ribosome scans the mRNA sequence from 5’ to 3’ until it reads an ATG codon with an appropriate context. If the AUG codon has an appropriate context, the translation initiates at that site and terminates when a stop codon is read. An in-frame codon is represented by three consecutive nucleotides that are grouped together. Part (a) of the figure presents an example of extraction of positive sequences (TIS) and parts (b) and (c) present out of frame and in frame negative sequences, respectively.

The sequences were extracted only from files containing the minimum number of nucleotides in the upstream region of the window. Thus, all the files that did not contain this number were disregarded. Having extracted fragments of negative sequences from the database, there were two possible classifications in accordance with the alignment of the ATG with the TIS: in frame or out of frame. If a sequence is in frame with the TIS, this means that it is aligned with the TIS. In other words, the start of the ATG is a position which is a multiple of 3 of the upstream and downstream regions with regard to the TIS. Figure 1 presents examples of extraction of positive and negative sequences given a molecule of mRNA. The TIS is determined by ATG, highlighted in red, and is represented by positions +1, +2 and +3. Figure 1 (a) presents an example of a positive sequence. Parts (b) and (c) of Figure 1 present examples of out of frame and in frame negative sequences, respectively.

In this study, two approaches for the extraction of positive and negative sequences were considered. In the first, all sequences that had ATG and were not the TIS were considered negative. The second approach, called **I**nclusion of **A**cquired **Know**ledge (InAKnow), considered that all ATGs that are in the downstream region had no classification, at first. This is considered since these ATGs are not evaluated by the *Ribosome Scanning Model* (RSM).

Table 1 presents the total number of mRNA molecules for the organisms (*Mus musculus* and *Rattus norvegicus*) in addition to the number of positive sequences (POS) and the out of frame negative sequences in upstream and downstream regions for the two approaches used (with or without InAKnow). A window size of -10+30 was used since this was the window that gave the best results.

**Table 1:** Number of positive, out of frame upstream and downstream negative sequences (OFN) with a window size of -10+30. Compares the two approaches: with and without the inclusion of the acquired knowledge method (InAKnow).

	Without InAKnow			With InAKnow		
	Positives	Up. Negatives	Down. Negatives	Positives	Up. Negatives	Down. Negatives

*Mus musculus*	269	327	5929	1063	327	4866
*Rattus norvegicus*	101	305	12940	379	305	12662

Total quantity of mRNA for organisms *Mus musculus* and *Rattus norvegicus* are respectively 309 and 1317. Download in 05/03/2011.

From this table, it should be observed that the problem is highly imbalanced, justifying investment in balancing methods. It should also be noted that the number of positive sequences extracted is not equal to the number of mRNA molecules, since only those sequences that had CDS greater than or equal to 10 nucleotides (the size of the upstream region) were extracted (94.5% and 8.66% for *Mus musculus* and *Rattus norvegicus*, respectively). Additionally, some molecules were discarded as they did not start with the ATG codon (7.44% for *Mus musculus* and 0.99% for *Rattus norvegicus*). The problem of class imbalance applied to all other window sizes analyzed.

In accordance with the main authors in the literature, the sequences were codified using the 4 bits codification scheme mentioned in the review of the current state of research.

### Class balancing

In the field of classification, a database is described as imbalanced when there are much fewer cases of some classes than others [22]. This type of problem is of great importance since datasets with this characteristic can be found in many areas. Many learning systems assume that classes are balanced and, as a result, these systems fail to produce a classifier that is capable of accurately predicting the minority class in the presence of data containing imbalanced classes [23]. Very frequently, the classifiers tend to value predominant classes (cases) and ignore the least frequent classes [24].

The problem of predicting the TIS is inherently imbalanced since a molecule of mRNA has only one ATG that codifies protein, while all the others are non-TIS. For the organisms *Mus musculus* and *Rattus norvegicus*, for example, there is an average disproportion of 1:23 and 1:131, respectively. This disproportion is 1:24, 1:51, 1:22, 1:22 and 1:10 for *Arabidopsis thaliana*, *Caenorhabditis elegans*, *Drosophila melanogaster*, *Homo sapiens* and Nasonia vitripennis, respectively.

It is worth noting that the problem would be even greater if evaluation of TIS was performed at the DNA level since the imbalance in this case would be far greater than at the mRNA level.

The sampling methods for class balancing aim to alter the distribution of the training data in order to increase the accuracy of its models [24]. This is achieved by eliminating cases of the majority class (undersampling) or replicating cases of the minority class (oversampling). In the literature, these are known as random undersampling and oversampling methods that do not use heuristics in the elimination/replication of cases and those that do [13,23-25].

According to Batista *et. al *[23], various authors agree that sampling methods that do not use heuristics can cause unwanted disturbances in the models generated. The simple replication of minority class cases can cause overfitting, while the random elimination of majority class cases can remove important information for the learning process.

In this study, the following balancing methods were used:

• ***Random undersampling*** This method randomly eliminates majority class cases with the aim of matching the quantity of minority class cases. It is used in this study to evaluate and validate the other methods used and proposed.

• ***SBC* (*Sampling Based on Clustering*)** A method of undersampling proposed by Yen and Lee [25] where the main idea is that there are different clusters in the database with different features. The complete database, composed of the minority and majority classes, is grouped into *k* clusters. From these clusters, samples of the majority class are selected according to the proportion of samples of this class (*SizeMA*) and the minority class (*SizeMI*) in each cluster *i*. The number of samples of the majority class selected in cluster *i*, represented by , is calculated by Equation 1..(1)

where *m* × *Size_MI_* is the total majority class samples selected that should be in the final training file and *m* indicates the proportion between the majority and minority classes; in this case 1:1.  is the total number of majority class samples to the number of minority class samples in all clusters. Thus, Equation 1 determines that more majority class samples would be selected in the cluster which behaves more like the majority class samples. In this study, the k-means clustering method was used with 4 clusters, a quantity already evaluated by Yen and Lee [25].

**• *M- Clus* (*Majority class undersampling based on Clustering*)** The main characteristic of the method proposed in this study is the creation of a clustering with the sequences of the majority class. From this clustering, the most significant characteristics of each cluster are selected for the training stage. Clustering is an unsupervised classification of patterns (observations, items of data or vectors of characteristics) in groups. Intuitively, each group is composed of patterns that are similar to each other and dissimilar to the patterns of other groups [26].

In order to create the clustering, the k-means algorithm proposed by Macqueen [27] was used, and applied to situations in which all of the variables are quantitative and the dissimilarities between them can be measured in a Euclidean distance [28].

The algorithm begins with the choice of the k elements that form the initial seeds. This choice can be made, among other methods, by selecting the first *k* observations, in a completely random manner or even in such a way that its values are very different.

Once the initial seeds are chosen, the distance of each element in relation to the seeds is calculated. The element is placed in the group that has the least distance (most similar) and the centroid is recalculated. The process is repeated until all of the elements are part of one of the clusters. After grouping all of the elements, an attempt is made to find a partition better than one generated arbitrarily. To this end, the degree of internal homogeneity of the groups is calculated using the Residual Sum of Squares (RSq) which is the measure used to evaluate the quality of a partition. After the calculation, the first object is moved to the other groups and verified for an increase or decrease in the value of the RSq. If there is a change, the object is moved to the group that generated the largest increase. The RSq of the groups is then recalculated and the process moves to the next object. After a certain number of iterations or when there are no further changes, the process is interrupted [27].

For the purpose of balancing, the quantity of clusters k varied between the total (*k_Size_MI__*), half (*k*_*Size_MI_*/2_) and one third (*k*_*Size_MI_*/3_) of the number of minority class sequences; and for each cluster, one, two and three sequences are removed, respectively. In order to select the sequences, those with the smallest distance to the centroid of the cluster are removed.

### Inclusion of features

In this study, in addition to its own sequence, some features reported in previous studies were included. Thus, by generating the training and test sets, a combination was formed between the extracted sequences and the selected features: presence or absence of an ATG upstream in frame with the TIS, presence or absence of a stop codon in the following 100 nucleotides [1,4,15,29,30], presence or absence of the codons CTG, GAC, GAG and GCC in the downstream region in frame with the TIS [4,15,29].

An ATG upstream in frame can be explained by the ribosome scanning model, which scans from region 5’ to region 3’ until it finds the first ATG which contains a translation context. Thus, an ATG closer to region 5’ has a high probability of being the TIS. Consequently, the presence of an ATG in the upstream region in frame with the TIS could indicate that the initially predicted TIS has less chance of being the actual TIS. This fact was also reported by Rogozin *et. al *[31] who demonstrated that a negative correlation exists between the quality of the context of the initiation of translation and the number of ATGs in the upstream region. This characteristic proved to be of great relevance to this study since the best results were obtained when this characteristic was included and combined with others.

The presence or absence of stop codons (TAA, TAG and TGA) in frame in the downstream region in the following 100 nucleotides is explained by the biological process of translating the in frame codons into amino acids. The translation process ends when a stop codon in frame is found. Therefore, the presence of any in frame stop codon in the following 100 nucleotides indicates that the protein should not have more than 33 amino acids. This is less than the majority of existing proteins, indicating that the ATG may not be the TIS [2].

Some features presented in previous studies, such as positions -3 and +4 and the size of the upstream and downstream regions, were not considered as they are implicit in the extracted sequences.

### Support vector machines

Support Vector Machines (SVM) is a machine learning technique founded on the inductive principles of Structural Risk Minimization. These principles originate from Statistical Learning Theory [32]. Characterized as a machine learning algorithm capable of resolving linear and nonlinear classification problems, the main idea of classification by support vector is to separate examples with a linear decision surface and to maximize the margin of separation between the other training points [2].

The SVM works as follows: Given a set of training data *x_i_*, , each with an input vector *x_i_* ∈ ℜ*^n^* and corresponding binary output *y_i_* ∈ [–1, +1], the objective is to separate the class -1 vectors from the class +1 vectors.

The SVM-light version implemented by T. Joachims [33], available at http://svmlight.joachims.org, was used here. A 4th order polynomial function was adopted and the trade-off between training error and margin was 1.0, defined by testing tirelessly (not shown).

### Inclusion of acquired knowledge

As shown in Figure 1, the scanning model supposes that the ribosome is first connected to region 5’ of the mRNA and travels in the direction of region 3’ until it finds the first ATG of the sequence [5]. However, there are exceptions: as a result of poor context (for example, noise), the first ATG could be ignored. Considering this ribosome scanning model, it can be observed that only the ATGs in the upstream region of the TIS and the TIS itself have classification. Thus, all other ATGs that are in the downstream region, a priori, has no definite classification. That is, there may be an appropriate context for ATG with TIS in the region downstream, and this is not only because the ribosome have already started the translation into an ATG before. Figure 1 presents this model: the ribosome, not identifying the first ATG(s) of the sequence as the TIS, moves to the second, third or more ATGs, classifying them as non-TIS until it finds the ATG that it classifies as TIS. In this sense, there is no exact classification for any of the ATGs in the downstream region of the TIS [2].

**Figure 2:**
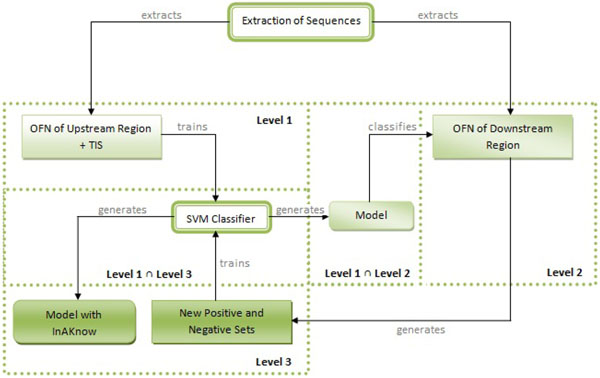
**Methodology of inclusion of acquired knowledge (InAKnow).** According to the ribosome scanning model, the sequences extracted from the downstream region of the TIS do not have classification. Thus, at Level 1 of this proposed methodology, the sequences of the upstream region (positive and negative) are trained by the classifier, and at Level 2 the model created by this training is applied to the sequences of the downstream region (without classification). Subsequently, the sequences of the downstream region (now classified), together with those of the upstream region are again inserted into new training, thereby generating a new model (Level 3).

Taking this into account, this study presents a methodology that uses the model created from the negative sequences of the upstream region and the positive sequences (TIS) to classify the negative sequences in the downstream region. Based on the classification obtained by this model, these sequences are classified and a new model is trained, thus obtaining the final model. In this new model, the number of positive sequences may increase since the system can identify a candidate TIS in the downstream region. This process of including acquired knowledge (InAKnow) is shown in Figure 2.

In accordance with this methodology, the following steps are followed:

1. Obtain a model, considering only the positive sequences (TIS) and the out of frame (OFN) negative sequences (non-TIS) contained in the upstream region (Figure 2 - Level 1);

2. Classify the ATGs in the downstream region using the model generated in the previous step (Figure 2 - Level 2);

3. New training with all of the sequences, including those classified by the previous step (Figure 2 -Level 3). In this stage, there is a decrease in the class imbalance due to the inclusion of sequences classified as positive by the model. Thus, the proportion of 1:23 was reduced, approximately, to 1:1 (*Mus musculus*) and 1:3 (*Rattus norvegicus*). The disproportion was reduced to 1:8, 1:7, 1:5, 1:5 and 1:1 for *Arabidopsis thaliana*, *Caenorhabditis elegans*, *Drosophila melanogaster*, *Homo sapiens* and *Nasonia vitripennis*, respectively.

### Measures of performance

Five measures were used to evaluate the performance of the classifier: accuracy (Ac), precision (Pr), sensitivity (Se), specificity (Sp) and adjusted accuracy (Acj) [2,4,10,15,29].

Accuracy measures the proportion of correct predictions, as described in Equation 2.(2)

where TP, TN, FP and FN denote the number of true positives, true negatives, false positives and false negatives, respectively.

Precision measures the proportion of possible TIS that are definitely TIS (Equation 3).(3)

Sensitivity, also known as the true-positive rate, refers to the percentage of correct items within the positive class. In other words, it measures the proportion of TIS that were correctly classified as TIS (Equation 4).(4)

Specificity, also known as the true-negative rate, refers to the percentage of correct items within the negative class. In other words, it measures the proportion of non-TIS that was correctly classified as non-TIS (Equation 5).(5)

Adjusted accuracy is the average of the sensitivity and specificity measures (Equation 6).(6)

All results presented in the “Results and Discussion” Section are based on these measures using the concept of cross validation. In addition to these performance measures, an alternative method for assessing the performance of these classifiers is the analysis of ROC curves [34]. An ROC graph can be used to analyze the relationship between false negatives (FN) and false positives (FP) or true negatives (TN) and true positives (TP) for a given classifier.

### Validation

10-fold cross validation was used, identified by Kohavi [35] as the most efficient form of evaluation for selecting models. The process of cross validation used in this study followed the methodology suggested by Machado [24], where the imbalanced database is initially divided into ten subsets. Nine subsets are reserved for training while only one is destined for testing. The training set is then balanced by the application of the balancing methods described above. This data is used in the SVM during training and tested with the reserved subset. This process is repeated ten times and after the final repetition, the average performance and standard deviation are calculated.

## Results and discussion

### Evaluation of the window size

**Figure 3:**
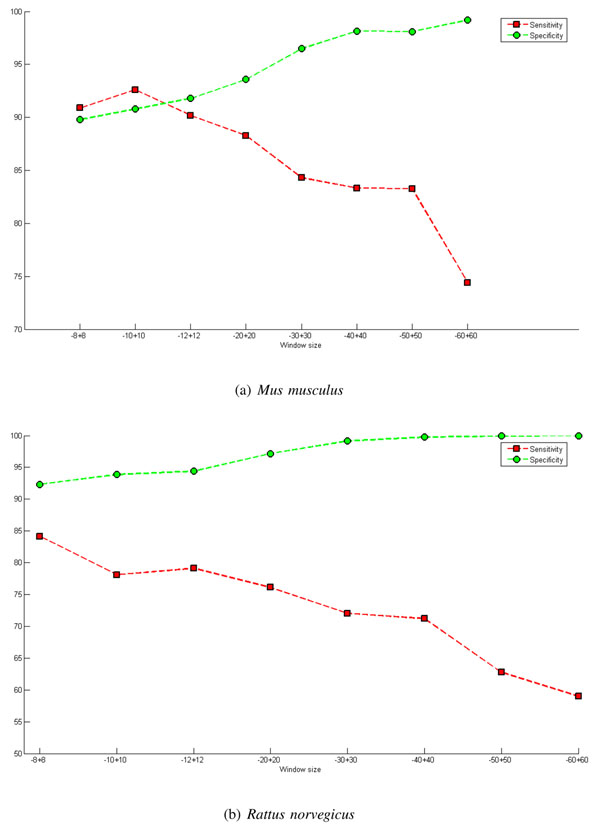
**Graph of window size - symmetrical.** Presents the results, particularly the rates of sensitivity and specificity, for various symmetrical window sizes. These results were obtained using the M-Clus balancing method and the features ATG + STOP + GAG. The inclusion of acquired knowledge, InAKnow, was not used. Parts (a) and (b) of the figure present results for *Mus musculus* and *Rattus norvegicus*, respectively.

Considering the databases of *Mus musculus* and *Rattus norvegicus*, extensive experiments were conducted with the aim of evaluating the size of window which offers the best performance. Tables 2 and 3 shows the results os this tests. The numbers between brackets are the corresponding standard deviations. Observing these tables, it can be observed that as the size of window increases there is an increase in the accuracy and specificity rates. On the other hand, there is a fall in sensitivity (this is an important measure). Increasing the window size in the upstream and downstream region at the same time causes the sensitivity and specificity rates to counter each other. In other words, when one increases, the other decreases, as shown in Figure 3 (a) and (b).

**Table 2:** Comparison of performance as a function of window size for the *Mus musculus* organism.

*Organism: Mus musculus*					
**Window size**	**Ac**	**Pr**	**Se**	**Sp**	**Adj**

**Evaluation of windows of symmetric size**					

-8+8	87,77 (1,50)	22,46 (2,41)	79,82 (9,16)	88,11 (1,69)	83,97 (4,25)
-12+12	91,35 (1,15)	29,91 (3,87)	81,13 (7,56)	91,77 (1,11)	86,45 (3,90)
-20+20	94,03 (0,69)	39,08 (4,15)	81,06 (5,96)	94,58 (0,66)	87,82 (3,03)
-30+30	96,42 (0,61)	54,08 (5,68)	81,99 (9,10)	97,03 (0,63)	89,51 (4,48)
-40+40	97,49 (0,55)	66,18 (7,68)	77,06 (6,66)	98,32 (0,63)	87,69 (3,22)
-50+50	98,21 (0,41)	77,95 (6,78)	74,17 (8,29)	99,16 (0,30)	86,66 (4,15)
-60+60	98,27 (0,53)	82,74 (4,66)	68,20 (11,75)	99,45 (0,18)	83,83 (5,86)

**Evaluation of windows of asymmetric size**					

*Evaluation of upstream region*					

-8+30	94,35 (0,89)	41,07 (5,44)	82,03 (8,65)	94,87 (0,96)	88,45 (4,22)
-10+30	**95,23** (1,09)	**46,44** (9,42)	**82,13** (8,94)	**95,79** (1,12)	**88,96** (4,48)
-12+30	94,77 (0,89)	43,99 (10,75)	81,28 (9,13)	95,29 (1,19)	88,28 (4,44)
-30+30	96,42 (0,61)	54,08 (5,68)	81,99 (9,10)	97,03 (0,63)	89,51 (4,48)
-50+30	97,39 (0,60)	63,65 (10,54)	78,45 (8,34)	98,14 (0,67)	88,30 (4,08)

*Evaluation of downstream region*					

-10+10	89,96 (1,35)	27,01 (3,19)	82,80 (7,96)	90,26 (1,60)	86,53 (3,51)
-10+20	92,44 (1,25)	33,49 (4,60)	81,85 (8,79)	92,90 (1,43)	87,37 (4,13)
-10+30	**95,23** (1,09)	**46,44** (9,42)	**82,13** (8,94)	**95,79** (1,12)	**88,96** (4,48)
-10+50	96,29 (0,98)	54,67 (10,78)	78,77 (7,13)	97,04 (1,09)	87,90 (3,47)

These results were obtained using class balancing and the M-Clus method. No features were considered and the method of including acquired knowledge, InAKnow, was not used.

**Table 3:** Comparison of performance as a function of window size for the *Rattus novergicus* organism.

*Organism: Rattus novergicus*					
**Window size**	**Ac**	**Pr**	**Se**	**Sp**	**Adj**

**Evaluation of windows of symmetric size**					

-8+8	92,28 (0,85)	7,77 (1,69)	84,17 (15,04)	92,34 (0,88)	88,25 (7,39)
-12+12	94,27 (1,09)	9,75 (1,67)	79,09 (15,84)	94,39 (1,16)	86,74 (7,58)
-20+20	96,99 (0,97)	19,06 (9,96)	76,11 (21,38)	97,16 (1,10)	86,63 (10,39)
-30+30	98,95 (0,28)	39,69 (10,19)	72,05 (17,56)	99,15 (0,32)	85,60 (8,72)
-40+40	99,56 (0,20)	72,17 (16,36)	71,21 (12,16)	99,76 (0,18)	85,49 (6,07)
-50+50	99,67 (0,18)	89,40 (11,72)	62,75 (17,34)	99,94 (0,06)	81,35 (8,66)
-60+60	99,70 (0,12)	94,67 (8,19)	59,54 (11,39)	99,97 (0,04)	79,76 (5,70)

**Evaluation of windows of asymmetric size**					

*Evaluation of upstream region*					

-8+30	97,07 (0,97)	20,40 (6,78)	83,17 (11,21)	95,15 (0,98)	89,16 (5,56)
-10+30	**97,14** (0,76)	**19,35** (4,36)	**82,09** (14,05)	**97,26** (0,79)	**89,67** (6,91)
-12+30	98,70 (0,52)	24,72 (7,45)	76,47 (16,50)	98,70 (0,43)	87,58 (8,05)
-30+30	98,95 (0,28)	39,69 (10,19)	72,05 (17,56)	99,15 (0,32)	85,60 (8,72)
-50+30	99,65 (0,18)	84,63 (11,14)	64,00 (16,81)	99,91 (0,08)	81,95 (8,39)

*Evaluation of downstream region*					

-10+10	93,76 (1,16)	8,94 (1,45)	78,09 (14,08)	93,88 (1,22)	85,99 (6,73)
-10+20	95,21 (0,85)	11,75 (2,11)	80,09 (11,04)	95,32 (0,87)	87,70 (5,46)
-10+30	**97,14** (0,76)	**19,35** (4,36)	**82,09** (14,05)	**97,26** (0,79)	**89,67** (6,91)
-10+50	98,80 (0,27)	37,76 (7,45)	78,09 (17,84)	98,97 (0,24)	88,53 (8,92)

These results were obtained using class balancing and the M-Clus method. No features were considered and the method of including acquired knowledge, InAKnow, was not used.

**Figure 4:**
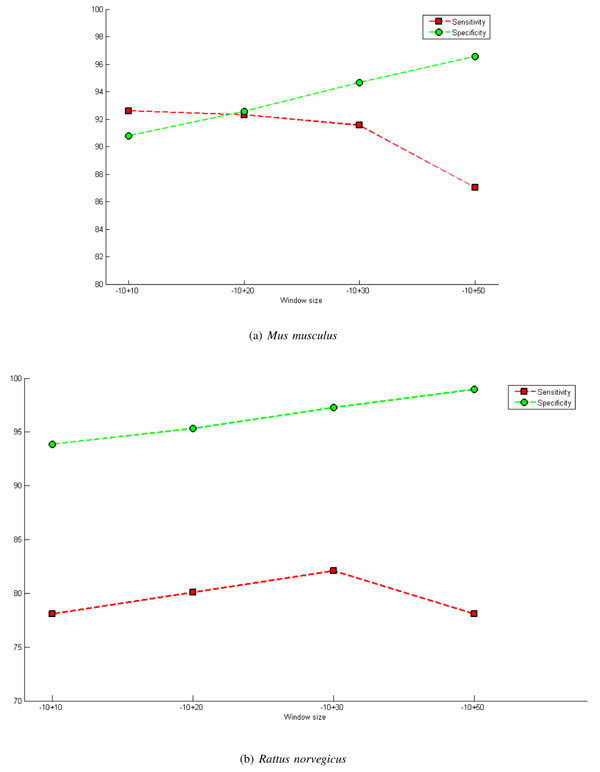
**Graph of window size - downstream region.** Presents the results, particularly the rates of sensitivity and specificity, for various asymmetrical window sizes where the downstream region is varied. These results were obtained using the M-Clus balancing method and the features ATG + STOP + GAG. The inclusion of acquired knowledge, InAKnow, was not used. Parts (a) and (b) of the figure present results for *Mus musculus* and *Rattus norvegicus*, respectively.

In an attempt to avoid this effect and improve the performance of the classifier, windows of asymmetrical sizes were exploited. From Figure 4 (a) and (b), it can be observed that increasing the size of the downstream regions results in an increase in specificity and a decrease in sensitivity; consequently, the size of this region should not be very large so as not to interfere with the rate of sensitivity but should not be too small to guarantee a good rate of specificity.

**Figure 5:**
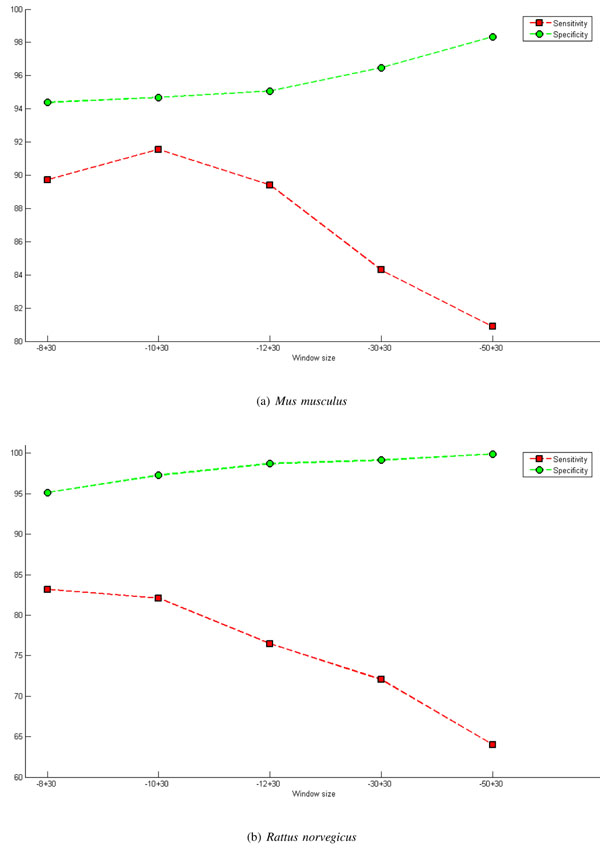
**Graph of window size - upstream region.** Presents the results, particularly the rates of sensitivity and specificity, for various asymmetrical window sizes where the upstream region is varied. These results were obtained using the M-Clus balancing method and the features ATG + STOP + GAG. The inclusion of acquired knowledge, InAKnow, was not used. Parts (a) and (b) of the figure present results for *Mus musculus* and *Rattus norvegicus*, respectively.

On the other hand, when there is an increase in the upstream region, there is a significant decrease in sensitivity (Figure 5 (a) and (b)). Thus, there is evidence that sensitivity is related to the nucleotides in positions close to the TIS. In other words, the context in which the ribosome initiates translation in a given ATG are the nucleotides before and after the ATG that is being validated. This corroborates the study of Hatzigeorgiou [6] which used the *ANN Consensus Model* with a window size of 12 nucleotides, from -7 to +5, and verified that this model was sensitive to the stable region of the TIS. Tzanis *et. al *[15] also used a component which analyzed the region around the TIS, from -7 to +5, using Markov chains to capture the consensus pattern, indicating that for the identification of the TIS it is important to examine a restricted area around it.

Among the tests conducted, the window size of -10+30 generated the best results, improving the adjusted accuracy, represented by the average of the sensitivity and specificity.

### Evaluation of the features included

From Tables 4 and 5, it can be observed that the inclusion of features improves the performance of the classifier. A comparison of the tests with no features with those which included ATG + STOP + GAG, reveals an increase of approximately 9.42% and 6% in the rate of sensitivity observed for *Mus musculus* and *Rattus norvegicus*, respectively. Moreover, the rate of specificity did not vary much at approximately 1.11% and 0.72% for *Mus musculus* and *Rattus norvegicus*, respectively.

**Table 4:** Comparison of performance as a function of the inclusion of features for the *Mus musculus* organism.

Features	Ac	Pr	Se	Sp	Adj
No	95.23 (1.09)	46.44 (9.42)	82.13 (8.94)	95.79 (1.12)	88.96 (4.48)
ATG + STOP + GAG	94.54 (1.15)	43.05 (6.18)	**91.55** (3.76)	94.68 (1.14)	93.11 (2.13)
ATG+STOP+CTG+GAC+GAG	94.48 (1.11)	42.69 (4.85)	**90.88** (4.80)	94.64 (1.20)	92.76 (2.27)
ATG + STOP	94.54 (0.89)	42.83 (5.44)	**90.59** (4.56)	94.72 (0.95)	92.66 (2.26)
ATG + STOP + CTG + GAC	94.54 (1.40)	43.35 (6.77)	**90.30** (4.91)	94.72 (1.45)	92.51 (2.53)
ATG+STOP+CTG+GAC+GAG+GCC	94.54 (0.99)	42.72 (4.76)	89.44 (5.08)	94.77 (0.98)	92.11 (2.66)
ATG	94.85 (1.28)	45.02 (8.66)	89.34 (4.38)	95.09 (1.37)	92.21 (2.18)
ATG + STOP + GCC	94.54 (1.18)	43.02 (6.56)	89.34 (5.01)	94.77 (1.23)	92.06 (2.55)
ATG + STOP + GAC	94.66 (1.34)	43.89 (7.62)	88.86 (4.37)	94.91 (1.41)	91.89 (2.20)
ATG + STOP + CTG	94.70 (1.17)	43.68 (5.90)	88.57 (4.12)	94.96 (1.20)	91.77 (2.18)
ATG + CTG	94.86 (1.18)	44.65 (6.69)	88.47 (3.44)	95.14 (1.23)	91.81 (1.79)
ATG + GAG	94.70 (1.41)	44.25 (8.95)	88.09 (4.69)	94.98 (1.45)	91.53 (2.49)
ATG + GCC	95.12 (1.11)	46.03 (6.78)	88.00 (5.92)	95.44 (1.14)	91.72 (3.02)
ATG + GAC	95.26 (0.78)	46.42 (5.63)	87.42 (5.41)	95.60 (0.81)	91.51 (2.70)
STOP + GAC	94.48 (1.31)	42.31 (7.73)	85.01 (8.51)	94.88 (1.19)	89.95 (4.52)
STOP + CTG	94.25 (1.35)	41.52 (7.51)	84.72 (5.71)	94.66 (1.42)	89.69 (2.88)
STOP	94.08 (1.23)	40.13 (7.01)	82.61 (8.31)	94.58 (1.19)	88.60 (4.30)
STOP + GAG	94.48 (1.49)	42.61 (8.69)	82.33 (8.27)	95.01 (1.48)	88.67 (4.30)
GCC	94.74 (1.14)	43.52 (7.86)	82.13 (8.77)	95.28 (1.22)	88.71 (4.31)
CTG + GAG	94.80 (1.13)	44.05 (8.56)	81.94 (7.69)	95.36 (1.21)	88.65 (3.80)
CTG + GAC + GAG	94.53 (1.11)	42.20 (6.34)	81.94 (9.26)	95.08 (1.14)	88.51 (4.60)
STOP + GCC	93.97 (1.45)	40.05 (7.72)	81.94 (7.69)	94.50 (1.60)	88.22 (3.70)
GAG	94.78 (1.14)	43.85 (8.45)	81.56 (9.38)	95.36 (1.23)	88.46 (4.63)
CTG	94.74 (1.36)	43.96 (9.04)	81.56 (9.22)	95.31 (1.49)	88.44 (4.50)
GAC	94.85 (1.13)	44.03 (7.79)	81.46 (8.75)	95.42 (1.10)	88.44 (4.45)
GAG + GCC	94.88 (1.34)	44.33 (8.70)	81.26 (8.89)	95.46 (1.23)	88.36 (4.70)
GAC + GAG	94.95 (1.10)	44.77 (8.06)	81.17 (9.00)	95.55 (1.21)	88.36 (4.39)
CTG + GAC	95.00 (1.11)	45.00 (8.98)	81.08 (8.16)	95.60 (1.15)	88.43 (4.11)
CTG + GAC + GAG + GCC	94.77 (1.22)	43.62 (8.15)	80.98 (8.27)	95.36 (1.18)	88.17 (4.28)
CTG + GCC	94.39 (1.34)	41.82 (8.03)	80.98 (8.45)	94.96 (1.41)	87.97 (4.18)
GAC + GCC	94.34 (1.06)	41.07 (6.78)	80.21 (9.11)	94.95 **(1.11)**	87.58 (4.50)

These results were obtained using a window size of -10+30 and the M-(Clus method. The method of including acquired knowledge, InAKnow, was not used.

**Table 5:** Comparison of performance as a function of the inclusion of features for the *Rattus novergicus* organism.

Features	Ac	Pr	Se	Sp	Adj
No	97,14 (0,76)	19,35 (4,36)	82,09 (14,05)	97,26 (0,79)	89,67 (6,91)
ATG + STOP + GAG	95,38 (1,09)	13,54 (3,65)	**88,09** (9,82)	96,54 (1,13)	92,32 (4,61)
ATG + STOP	95,64 (0,82)	13,83 (2,18)	**88,09** (8,74)	95,69 (0,87)	91,89 (4,08)
ATG+STOP+CTG+GAC+GAG	96,48 (0,78)	16,93 (3,81)	**88,09** (9,88)	95,44 (0,89)	91,76 (4,35)
ATG + STOP + CTG + GAC	95,03 (1,19)	12,68 (2,94)	**88,09** (9,44)	95,08 (1,20)	91,58 (4,75)
ATG+STOP+CTG+GAC+GAG+GCC	96,38 (0,63)	16,06 (2,34)	87,18 (8,87)	96,45 (0,64)	91,82 (4,40)
ATG + GAC	96,57 (0,74)	17,09 (3,55)	87,09 (10,08)	96,64 (0,76)	91,86 (4,93)
ATG + CTG	96,42 (0,53)	16,22 (1,72)	87,09 (9,82)	96,49 (0,58)	91,79 (4,76)
STOP + GAC	96,30 (0,82)	16,08 (3,59)	87,09 (10,08)	96,37 (0,84)	91,73 (4,97)
ATG + STOP + GCC	96,11 (0,61)	15,52 (2,19)	87,09 (8,30)	96,15 (0,63)	91,62 (4,04)
ATG + STOP + GAC	95,68 (1,24)	14,55 (3,72)	87,09 (8,74)	95,73 (1,28)	91,41 (4,03)
ATG + STOP + CTG	94,97 (1,16)	12,54 (2,90)	87,09 (9,45)	95,02 (1,18)	91,05 (4,70)
ATG + GAG	96,70 (0,61)	17,54 (3,11)	86,09 (10,79)	96,76 (0,62)	91,42 (5,35)
ATG	96,32 (0,83)	15,97 (3,29)	86,09 (9,21)	96,40 (0,84)	91,24 (4,54)
STOP	95,99 (0,81)	14,62 (2,50)	86,09 (12,03)	96,07 (0,84)	91,08 (5,88)
ATG + GCC	96,93 (0,56)	18,05 (2,71)	84,09 (12,85)	97,02 (0,61)	90,56 (6,25)
STOP + CTG	95,86 (0,86)	14,04 (2,97)	84,09 (13,61)	95,95 (0,89)	90,02 (6,70)
STOP + GAG	96,36 (0,65)	15,51 (2,66)	84,09 (14,32)	96,45 (0,70)	90,27 (7,00)
CTG + GAC	96,84 (0,85)	17,90 (3,56)	83,09 (11,06)	96,94 (0,88)	90,01 (5,40)
GAG	97,35 (0,47)	20,13 (4,17)	82,09 (14,05)	97,46 (0,48)	89,78 (6,99)
GAC	96,99 (0,37)	17,82 (2,22)	82,09 (14,05)	97,10 (0,43)	89,59 (6,90)
GAG + GCC	91,65 (0,39)	22,00 (2,82)	81,18 (14,45)	97,77 (0,46)	89,48 (7,07)
CTG + GAG	97,25 (0,51)	19,32 (3,60)	81,18 (15,78)	97,37 (0,55)	89,28 (7,79)
CTG + GCC	97,47 (0,53)	21,04 (5,05)	81,09 (14,51)	97,59 (0,54)	89,34 (7,24)
GAC + GCC	97,54 (0,71)	21,73 (3,83)	80,09 (14,90)	97,67 (0,77)	88,88 (7,23)
GCC	97,52 (0,41)	20,74 (3,73)	80,09 (18,49)	97,65 (0,47)	88,87 (9,13)
GAC + GAG	97,50 (0,38)	20,79 (4,97)	80,09 (15,55)	97,63 (0,35)	88,86 (7,80)
CTG + GAC + GAG	97,05 (0,64)	18,25 (4,06)	80,09 (14,90)	97,18 (0,64)	88,64 (7,43)
CTG + GAC + GAG + GCC	97,52 (0,45)	20,75 (4,44)	79,09 (17,06)	97,66 (0,46)	88,37 (8,49)
STOP + GCC	96,91 (0,54)	17,15 (3,47)	79,09 (17,06)	97,05 (0,59)	88,07 (8,41)
CTG	96,69 (0,72)	16,20 (3,51)	78,18 (17,80)	96,83 (0,78)	87,50 (8,71)

These results were obtained using a window size of -10+30 and the M-Clus method. The method of including acquired knowledge, InAKnow, was not used.

Theses results were generated using the balancing method proposed in this study, M-Clus, and a window size of -10+30. These tests were also applied to other sizes of window, -50+50, -12+12 and -10+20, and this behavior was observed in all situations. Thus, the inclusion of features is relevant for increasing the sensitivity of the classifier.

However, there are features which, when added to the sequences, slightly decreased the performance of the classifier (from 0.19% to 1.92% for *Mus musculus* and from 0.91% to 3.91%, for *Rattus novergicus*). For example, for *Mus musculus*, adding the features CTG or GAG or GAG or CTG + GAG or GAC + GCC, causes a decrease of approximately 1.9% in the rate of sensitivity. For *Rattus novergicus*, adding the features CTG + GAC + GAC + GCC or STOP + GCC causes a decrease of approximately 3% in the rate of sensitivity. However, this variation is too small to be considered a decrease in the performance of the classifier.

Interestingly, the characteristics which gave the best performance by the classifier for the organism *Mus musculus* also gave the best performance when applied to the *Rattus norvegicus* organism. Table 6 presents the sixteen most important features, noting the sensitivity for the two organisms analyzed.

**Table 6:** The sixteen most important features for each organism.

Features	*Mus musculus*	*Rattus novergicus*
ATG + STOP + GAG	1	1
ATG+STOP+CTG+GAC+GAG	2	3
ATG + STOP	3	2
ATG + STOP + CTG + GAG	4	4
ATG+STOP+CTG+GAC+GAG+GCC	5	5
ATG	6	13
ATG + STOP + GCC	7	9
ATG + STOP + GAG	8	10
ATG + STOP + CTG	9	11
ATG + CTG	10	7
ATG + GAG	11	12
ATG + GCC	12	15
ATG + GAC	13	6
STOP + GAC	14	8
STOP + CTG	15	16
STOP	16	14

The following are the main features that were highlighted: ATG + STOP + GAG, ATG + STOP + CTG + GAC + GAG, ATG + STOP, ATG + STOP + CTG + GAC. In addition to this, the ATG upstream in frame characteristic is highly relevant since the best results were obtained by the combination of this with other features. It is worth emphasizing that there is a significant increase in sensitivity of 7.21% and 4% for *Mus musculus* and *Rattus norvegicus*, respectively, when only the ATG upstream characteristic is considered.

Thus, the tests conducted demonstrate that the classifier achieves good performance levels when it considers only the positive and negative sequences (linear sequence of bases). However, it also demonstrates that it is possible to increase performance by including features deemed relevant for the context considered.

### Evaluation of the balancing methods

**Figure 6:**
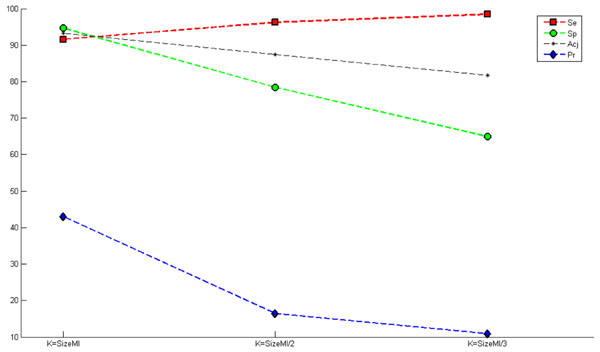
**Graph of the M-Clus balancing method.** Presents the results of the M-Clus method where the quantity of clusters created (*k*) is varied. These results were obtained using window size of -10+30 and the features ATG + STOP + GAG. The inclusion of acquired knowledge, InAKnow, was not used.

Figure 6 presents the results of the *M-Clus balancing method*, with variation in the number of clusters. It should be observed that when the number of clusters is decreased, the rates of specificity and precision decrease. This can be understood by the low representativeness of the negative class (majority), since when the quantity of clusters is decreased by half, two sequences are extracted per cluster. Thus, the sequences extracted are those closest to the centroid and, consequently, are close to each other. Therefore, there is no good representativeness for all negative sequences. Thus, to obtain greater representativeness of the majority class, the quantity of clusters was considered equal to the quantity of elements of the minority class (*SizeMI*).

A comparison of the results obtained by the other balancing methods analyzed, presented in Table 7, shows that when no balancing method is used the rate of sensitivity is very low at 51.39% for *Mus musculus* and 47.45% for *Rattus novergicus*. This occurs because the database is imbalanced and the classifier therefore learns a lot about the negative class (majority) and little about the positive class (minority). Thus, the system tends to find a large number of false negatives, and, through Equation 4, it can be observed that the sensitivity decreases with an increase in FN (false negatives).

**Table 7:** Comparison of performance as a function of the balancing method.

*Organism: Mus musculus*					
**Balancing**	**Ac**	**Pr**	**Se**	**Sp**	**Adj**

Without balancing	97,96 (0,37)	98,50 (3,02)	51,39 (6,97)	99,97 (0,06)	75,68 (3,49)
Rand undersampling	93,70 (0,83)	38,95 (3,94)	91,06 (3,85)	93,81 (0,88)	92,44 (1,90)
M-Clus	94,54 (1,15)	43,05 (6,18)	**91,55** (3,76)	**94,68** (1,14)	**93,22** (2,13)
SBC	92,23 (1,70)	34,12 (4,96)	89,63 (3,46)	92,34 (1,81)	90,98 (1,74)

*Organism: Rattus norvegicus*					

**Balancing**	**Ac**	**Pr**	**Se**	**Sp**	**Adj**

Without balancing	99,59 (0,08)	96,90 (6,21)	47,45 (11,21)	99,98 (0,03)	73,72 (5,60)
Rand undersampling	95,90 (2,07)	13,89 (4,57)	83,18 (10,75)	96,00 (2,36)	89,59 (4,89)
M-Clus	95,38 (1,09)	13,55 (3,65)	88,09 (9,82)	95,44 (1,14)	91,76 (4,61)
SBC	88,23 (6,09)	6,73 (2,57)	91,00 (11,35)	88,20 (6,20)	89,60 (4,09)

These results were obtained using a window size of -10+30, the features ATG + STOP + GAG. The inclusion of acquired knowledge, InAKnow, was not used.

This fact supports the authors Machado [24] when they state that the classifiers generated from imbalanced databases present high levels of false negatives for rare classes which is problematic when these are the classes being studied.

The use of any of the balancing methods analyzed increases sensitivity by around 40% for both *Mus musculus* and *Rattus norvegicus*. The method of balancing proposed, M-Clus, performed better than all other methods, especially with regard to the rate of adjusted accuracy. For the organism *Mus musculus*, the best performance compared to the rate sensitivity refers to the method M-Clus. As for the *Rattus norvegicus* best value for the rate sensitivity is given to using the SBC method, but there is a drop in rates of accuracy and specificity and hence in the adjusted accuracy. The random undersampling method is slightly better than the SBC method, its use being of interest because it is a simple method to implement. An analysis of the rate of precision reveals that the performance is 98.5% and 96.9% without the use of any balancing techniques. This rate is significantly reduced when a method is used to conduct the balancing. This can be explained by the fact that when no balancing method is used, the classifier learns little about the positive class. Consequently, few samples from the test set are classified as positive and few false positives are therefore generated. As the rate of precision evaluates how many possible TIS (classified as TIS) are actually TIS, this rate is of great value since few examples are classified as TIS and, consequently, few are false positives. In other words, as the precision is given by Pr = TP/(TP+FP), the rate increases with the reduction of FP (false positives).

Finally, it is important to emphasize the necessity of presenting all measures of performance since it is possible to have a system with a very high level of accuracy but which presents practically no knowledge with respect to the class of interest.

With the objective of improving the level of precision, a new methodology, described in the background section, was planned, namely the method of *including acquired knowledge*. The results are presented below.

### Evaluation of the method of including acquired knowledge

The use of the methodology of including acquired knowledge (InAKnow), described in the background section, increased all the rates evaluated, especially the rate of precision which increased by 39% (Table 8) for *Mus musculus*. The rates of sensitivity, specificity and adjusted accuracy increased by 1.68%, 1.33% and 1.51%, respectively. For *Rattus norvegicus*, the increase was 22.0%. However, the accuracy value was still low. This can be explained by the small amount of positive sequences (only 101) due to the fact that 91% of mRNA molecules were disregarded by initiating translation at positions prior to 10 nucleotides (window size of the upstream region).

**Table 8:** Comparison of performance with and without the inclusion of the acquired knowledge method (InAKnow).

	Without InAKnow					With InAKnow				
	Ac	Pr	Se	Sp	Adj	Ac	Pr	Se	Sp	Adj

*Mus musculus*	94,54	43.05	91.55	94.68	93.22	95.56	82.05	93.23	96.01	94.62
	(1.15)	(6.18)	(3.76)	(1.14)	(2.13)	(0.78)	(2.82)	(2.69)	(0.74)	(1.42)

*Rattus norvegicus*	95,38	13.54	88.09	96.54	92.32	94.90	35.63	95.24	94.89	95.07
	(1.09)	(3.65)	(9.82)	(1.13)	(4.61)	(0.80)	(3.36)	(3.78)	(0.89)	(1.67)

When analyzing the results of experiments for the organism *Rattus norvegicus*, a significant improvement in the rate of sensitivity (8.15%) was observed when using the InAKnow methodology, meaning that the classifier learned better from positive sequences. As for the rates of accuracy and specificity, there was a slight drop of 0.31% and 0.38%, respectively.

The significant improvement in the rate of precision is due to the reduction in the number of samples classified as false positive. This reduction occurs in accordance with the ribosome scanning model [5] which does not evaluate the ATGs in the downstream region of the TIS where there may be sequences of ATG with the appropriate context to be the TIS.

Using the proposed methodology, the sequences of the downstream region of the TIS are initially classified via a previously generated model, using only the known TIS sequences and the negatives from the upstream region and those which are out of frame. Only after this initial classification will these sequences form part of the final training and test sets.

The sequences that were in the downstream region but which possess the necessary features to be the TIS will form part of the positive sequence set since, as per the ribosome model, these sequences can become the TIS if no ATG with the appropriate context has been found. Using this methodology, 14% of the sequences that were in the downstream region were classified as positive for *Mus musculus* and 2.15% for *Rattus novergicus*. These rates were 7.5%, 10.9%, 13.1%, 13.1% and 47.62% for *Arabidopsis thaliana*, *Caenorhabditis elegans*, *Drosophila melanogaster*, *Homo sapiens* and *Nasonia vitripennis*, respectively. This methodology, therefore, is of fundamental importance for obtaining a classifier with a high rate of precision and demonstrates how the knowledge acquired by the classifier is relevant for classifying sequences with an unknown classification a priori.

**Figure 7:**
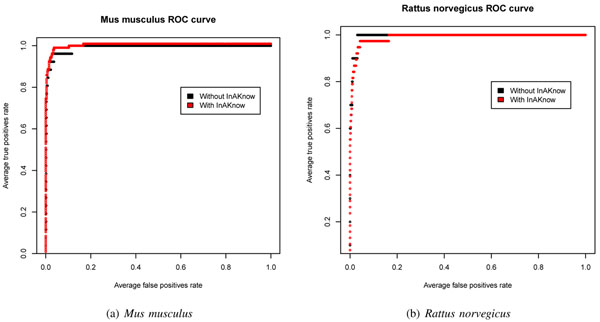
**ROC curve for *Mus musculus* and *Rattus norvegicus.*** Presents the ROC curve for *Mus musculus* and *Rattus norvegicus*, without and with the inclusion of the acquired knowledge methodology (InAKnow).

Figure 7 show the ROC curve, plotted in R [36], for *Mus musculus* and *Rattus norvegicus*, with and without the inclusion of the acquired knowledge methodology (InAKnow).

## Validation of the methodology with other databases

Once the methodology was fully tested for *Mus musculus* and *Rattus norvegicus* and the best settings for each of the tests was identified (window size, added features, balancing method and inclusion of knowledge), larger databases were also evaluated.

Thus, based on the best configuration obtained, the databases of the organisms *Aradidopsis thaliana*, *Caenorhabditis elegans*, *Drosophila melanogaster*, *Homo sapiens* and *Nasonia vitripennis*, which were extracted from the RefSeq database, were also validated. The number of positive, out of frame negative sequences in upstream and downstream regions are presented in Table 9, with and without the inclusion of the acquired knowledge Methodology (InAKnow).

**Table 9:** Number of positive, out of frame upstream and downstream negative sequences (OFN) with a window size of -10+30. Compares the two approaches: with and without the inclusion of the acquired knowledge method (InAKnow).

	Without InAKnow			With InAKnow		
	Positives	Up. Negatives	Down. Negatives	Positives	Up. Negatives	Down. Negatives

*Arabidopsis thaliana*	24339	17267	570619	68572	17267	526386
*Caenorhabditis elegans*	8763	6188	443052	57989	6188	393826
*Drosophila melanogaster*	19782	31269	404623	76907	31269	347498
*Homo sapiens*	15845	17495	336111	62212	17495	289744
*Nasonia vitripennis*	31	19	315	181	19	165

Total quantity of mRNA for organisms *Arabidopsis thaliana*, *Caenorhabditis elegans*, *Drosophila melanogaster*, *Homo sapiens* and *Nasonia vitripennis* are respectively 33200, 23894, 22303, 16264 and 35. Download in 05/03/2011.

Table 10 presents the results obtained with and without the inclusion of knowledge, using the random undersampling method.

**Table 10:** Comparison of performance without and with the inclusion of the acquired knowledge methodology (InAKnow).

	Without InAKnow					With InAKnow				
	Ac	Pr	Se	Sp	Adj	Ac	Pr	Se	Sp	Adj

*Arabidopsis thaliana*	91.52	31.68	91.94	91.49	91.72	92.78	68.88	75.29	94.98	85.13
	(1.88)	(4.46)	(1.71)	(2.02)	(0.33)	(2.42)	(11.00)	(8.41)	(3.66)	(2.64)

*Caenorhabditis elegans*	90.16	15.08	89.42	90.17	89.79	93.60	74.57	85.27	94.82	90.04
	(0.12)	(0.15)	(0.87)	(0.14)	(0.40)	(1.08)	(12.50)	(21.51)	(2.81)	(9.48)

*Drosophila melanogaster*	93.21	42.09	90.92	93.31	92.11	93.06	85.13	79.26	95.86	87.56
	(2.64)	(13.96)	(6.16)	(2.99)	(1.95)	(3.74)	(13.86)	(13.54)	(5.99)	(5.12)

*Homo sapiens*	93.53	42.15	91.93	93.60	92.76	93.31	89.00	72.97	97.43	85.19
	(2.70)	(10.84)	(2.96)	(2.94)	(0.60)	(2.34)	(9.91)	(22.26)	(2.83)	(10.03)

*Nasonia vitripennis*	85.00	83,64	100	53.00	76.5	87.5	88.64	96.67	73.00	84.83
	(16,66)	(19,67)	(0)	(47,75)	(23,88)	(16.77)	(20.02)	(10.00)	(41.96)	(20.50)

Although the M-Clus method offered a slightly better performance than the random method for *Mus musculus* and *Rattus norvegicus*, it requires greater computational time than other methods for clustering all sequences in the training file. Since the clustering was performed using the k-means algorithm with the Euclidean distance function, the distances of all sequences from possible initial centroids were calculated. *K* corresponds to the number of sequences from the minority class. Each time the centroids were modified, the distances of all sequences to the new centroids were also recalculated, searching k clusters with greater similarity between the sequences of the group and higher dessimilaridade between groups. This greatly increases the processing time for large databases.

Thus, as there is a significant delay in executing the M-Clus algorithm, and since, according to the results already presented, its performance is similar to the random method, we used the random undersampling method in conjunction with the knowledge inclusion method (InAKnow), which produced good results. In Table 10, it can be observed that the rates increased with the use of InAKnow, especially the precision which increased by 37.2%, 70.19%, 37.17%, 30.82% and 5% for *Aradidopsis thaliana*, *Caenorhabditis elegans*, *Drosophila melanogaster*, *Homo sapiens* and *Nasonia vitripennis*, respectively. That is, with InAknow, the model better learns the true positives since it demonstrates a higher rate of correctly identifying those which are truly positive.

This is probably due to the fact that the InAKnow method improves the knowledge of the model by recovering more positive sequences, thus yielding an increase in precision. These sequences are extracted from the downstream region and are assumed as negatives a priori.

However, sensitivity decreased most for *Homo sapiens* (18.96%), for interval confidence between 56.17% and 89.75 for a confidence level of 95%. Analyzing the results of each fold, we find that folds 1, 6 and 5 are the ones responsible for this decrease, as per Table 11. Further studies will be carried out to analyze these sequences added by InAKnow.

**Table 11:** Sensitivity, by fold (F), of the classifier using the methodology InAKnow.

	F1	F2	F3	F4	F5	F6	F7	F8	F9	F10
*Homo sapiens*	46.87	66.87	88.87	96.89	92.41	33.59	97.17	89.10	69.98	47.87

In general, the low sensibility can be attributed to two causes. Firstly, the model polarized the acquisition of knowledge because it adjusted to a larger number of negative than positive sequences. In the present approach, this situation does not occur due to the fact that the training set was balanced. Secondly, the training set contains false negative sequences. In both cases, parameter FN from equation 4 tends to increase, diminishing the value of sensitivity.

From examination of the *Mus musculus* and *Rattus norvegicus* databases, analyzed in Table 1, it is clear that they contain less positive sequences than negative sequences (upstream region). Thus, it is beneficial to increase the number of positive sequences through the InAKnow method. This increased the precision. Conversely, in Table 9, it is observed that there are some databases where the number of true positive sequences is larger than the number of true negative sequences (upstream region). Since the InAKnow method adds new sequences that have been identified as positives, which are extracted from the downstream region, the difference between the two types of sequences increases.

In this situation, which is less common (more positive than negative sequences), the present approach performs the balancing. That is, it increases the number of negative sequences using downstream sequences. It is important to note that it is not assumed that sequences in this region are all negatives. This can lead the model to increase the value of the false negative (FN) parameter and consequently decrease sensitivity. Taking this into account, the search for knowledge associated with the sequences that are not TIS seems important. We believe that the proposed InAKnow method can evolve, incorporating new knowledge that confirms that the downstream sequences are truly negatives. From this perspective, it would be possible to create acquired knowledge inclusion models that are more robust.

## Comparison with other TIS prediction tools

The methodology used in this study is compared with the First-ATG [5], NetStart [9] (available at http://www.cbs.dtu.dk/services/NetStart/), TIS Miner [37] (available at http://dnafsminer.bic.nus.edu.sg/Tis.html), and ATGpr (available at http://flj.hinv.jp/ATGpr/atgpr/index.html) programs.

The First-ATG method, proposed by Kozak (1984), proposes that the TIS of a molecule of mRNA is the first-ATG. For every molecule where TIS genuinely is the first-ATG, a TP (true positive) is added and each molecule where the TIS is not the first-ATG, a FP (false positive) is added.

To interpret the results reported by the Netstart tools, the methodology adopted by Sparks and Brendel [20] was used. Since this method is a TIS classifier and not a TIS prediction system, if the prediction given to the TIS is “Yes” (indicating that it TIS) a true-positive is counted. If it is not, a false negative is recorded. For every negative in the upstream region of the system set, the prediction is counted as a true-negative and false-positive results are not accounted for. The web interface and its Vertebrate-specific parameters were used. For the TIS Miner and ATGpr tools, the same methodology was used with the default settings.

Table 12 presents the results for the *Mus musculus* and *Rattus norvegicus* organisms. It can be observed that the methodology used in this study obtained a better performance by observing the sensitivity rate. The performance of the methodology used in this study for the organism *Rattus norvegicus* can be understood by the fact that for most of the sequences of this organism the TIS starts in the first position of the mRNA. These sequences are not analyzed by the methodology as they do not have 10 nucleotides in the upstream region of TIS (50.39% in total). Thus, many sequences were not analyzed by the methodology since these tests selected 20% of mRNA molecules in a random order.

**Table 12:** Comparison with other TIS prediction tools.

*Mus musculus*									
	TP	FP	TN	FN	Ac	Pr	Se	Sp	Adj

*First-ATG*	47	61	0	0	43.52	43.52	-	-	-
*NetStart*	72	21	113	15	83.71	77.42	82.76	84.33	83.54
*TIS Miner*	82	15	134	11	89.26	84.54	88.17	89.93	89.05
*ATGpr*	90	18	155	0	93.15	83.33	-	89.59	-
*Our methodology*	85	17	73	6	87.29	83.33	93.41	81.11	87.26

*Rattus norvegicus*									

	TP	FP	TN	FN	Ac	Pr	Se	Sp	Adj

*First-ATG*	97	30	0	0	76.38	90.65	-	-	-
*NetStart*	52	33	64	42	66.49	61.18	55.32	65.98	60.6
*TIS Miner*	53	73	75	1	63.36	42.40	98.15	50.67	74.71
*ATGpr*	100	27	78	0	86.83	78.74	-	74.28	-
*Our methodology*	32	34	52	0	71.19	48.48	-	60.46	-

All raw output generated by these tools on our test data is available as supplementary information at [21].

## Conclusions

As demonstrated in this study, the task of predicting the TIS is not a simple problem to resolve. Innumerable methods have been evaluated in the literature and this study presents a new methodology for finding the TIS based on balancing methods, including features and the concept of knowledge inclusion. What the authors aimed to do throughout the development of the study was to present methods which find TIS which are actually TIS. This was also done with a concern for the number of sequences used.

Since this problem is intrinsically imbalanced, undersampling class balancing methods were evaluated and the M-Clus undersampling method was also proposed. Undersampling methods, in contrast to oversampling methods which replicate the number of sequences, have the advantage of working with a much smaller number of sequences which appreciably reduces computational processing. This is particularly important in large databases like that of *Homo sapiens*, *Drosophila melanogaster*, *Arabidopsis thaliana* and *Caenorhabditis elegans*, analyzed in this study.

Considering the performance measures evaluated, the proposed balancing method proved to be very promising, offering the best results when compared to the random undersampling balancing method, SBC and classification without balancing. With M-Clus, there was an increase of 40.16% in the rate of sensitivity and 17.54% in the rate of adjusted accuracy, indicating that investment in balancing methods is necessary to resolve the problem. However, the precision was reduced by 55.45%, a problem which was resolved by the inclusion of acquired knowledge.

However, the proposed method also has a disadvantage since the number of interactions to get the best clusters demands a very large computational processing time. In larger databases this may be limiting and in this study, due to time constraints, the random balancing method was used to balance the larger databases since this was also efficient for the proposed problem. Two solutions to this problem are being worked on: 1) create heuristics to limit the number of iterations performed to obtain the best clusters, and 2) implement the methodology in a parallel and distributed manner, rather than sequentially.

There was an increase of up to 70.19% in precision when knowledge acquired (InAKnow) by the classifier was included in the new training set. This is due to the reduction in the number of samples classified as false positive in accordance with the ribosome scanning model [5] which does not evaluate the ATGs in the downstream region of the TIS where there may be sequences of ATG with the appropriate context to be the TIS.

The inclusion of certain features with the extracted sequences was also analyzed and it was concluded that, in general, this improves the performance of the classifier. The inclusion of features such as the presence of ATG in the upstream region of the TIS improved the rate of sensitivity by approximately 7.21% for *Mus musculus* and 4% for *Rattus norvegicus*.

Finally, according to the tests conducted on window size, there is evidence that sensitivity is related to the nucleotides close to the TIS. In other words, the context for the ribosome to initiate translation in a determined ATG are the nucleotides before and after the ATG that is being validated. The window that generated the best results had 10 nucleotides in the upstream region and 30 in the downstream region (-10+30).

In light of all the arguments presented, it is concluded that the methodology proposed contributes in a significant way to the prediction of the TIS.

## Competing interests

The authors declare that they have no competing interests.

## Authors' contributions

LS and FC have developed the methods and conducted the tests. CN, LZ and JM have provided the expertise and all three authors have drafted, read and approved the manuscript.
